# Prioritized experience replay in path planning via multi-dimensional transition priority fusion

**DOI:** 10.3389/fnbot.2023.1281166

**Published:** 2023-11-15

**Authors:** Nuo Cheng, Peng Wang, Guangyuan Zhang, Cui Ni, Erkin Nematov

**Affiliations:** ^1^School of Information Science and Electrical Engineering, Shandong Jiaotong University, Jinan, Shandong, China; ^2^Institute of Automation, Shandong Academy of Sciences, Jinan, China; ^3^Faculty of Mechanical Engineering, Tashkent State Technical University named after Islam Karimov, Tashkent, Uzbekistan

**Keywords:** deep reinforcement learning, deep deterministic policy gradient, prioritized experience replay, multi-dimensional transition priority, priority fusion

## Abstract

**Introduction:**

Deep deterministic policy gradient (DDPG)-based path planning algorithms for intelligent robots struggle to discern the value of experience transitions during training due to their reliance on a random experience replay. This can lead to inappropriate sampling of experience transitions and overemphasis on edge experience transitions. As a result, the algorithm's convergence becomes slower, and the success rate of path planning diminishes.

**Methods:**

We comprehensively examines the impacts of immediate reward, temporal-difference error (TD-error), and Actor network loss function on the training process. It calculates experience transition priorities based on these three factors. Subsequently, using information entropy as a weight, the three calculated priorities are merged to determine the final priority of the experience transition. In addition, we introduce a method for adaptively adjusting the priority of positive experience transitions to focus on positive experience transitions and maintain a balanced distribution. Finally, the sampling probability of each experience transition is derived from its respective priority.

**Results:**

The experimental results showed that the test time of our method is shorter than that of PER algorithm, and the number of collisions with obstacles is less. It indicated that the determined experience transition priority accurately gauges the significance of distinct experience transitions for path planning algorithm training.

**Discussion:**

This method enhances the utilization rate of transition conversion and the convergence speed of the algorithm and also improves the success rate of path planning.

## 1 Introduction

Intelligent robots have become increasingly diverse and essential across various industries due to robotics and artificial intelligence technology advancements. Examples include home-sweeping robots, shopping guide robots, and automatic sorting robots in the logistics industry. Path planning underpins intelligent robot motion and is a prevalent research topic. This process involves perceiving the environmental information through sensors, determining the robot's posture, and then identifying an optimal path from its current position to a goal location. Traditional path planning algorithms primarily encompass the Dijkstra algorithm (Liu L.-s. et al., 2021), the A^*^ algorithm (Hong et al., 2021), the ant colony algorithm (Miao et al., 2021), and enhancements based on these algorithms. Although these algorithms perform adequately in known environments, they struggle with convergence speed, operation time, and adaptation in unknown environments (Sang et al., 2021; Hou et al., 2022).

In recent years, deep reinforcement learning (DRL) has found applications in numerous fields (Lu et al., 2021; Wei et al., 2021; Zhu et al., 2023), and the path planning algorithm combined with DRL has gradually become a research hotspot. DRL-based path planning does not necessitate prior information about the environment. Instead, it predicts the next action based on sensing the current state. Once an action is executed, the robot receives the reward from the environment, facilitating its movement from the current state to the next state. This process is repeated until the robot reaches its target point or the maximum number of steps is achieved (Chen et al., 2021; Liu X.-h. et al., 2021; Sinha et al., 2022). Q-learning is a value-based reinforcement learning algorithm (Golowich and Moitra, 2022). For scenarios with small state-action spaces, states and actions can be stored in a dynamically changing Q-table for path planning. In each episode, suitable actions from the table are selected. However, as the environment of the robot becomes intricate and the movement area expands, the Q-table's capacity grows exponentially, increasing its search time and affecting the robot's learning efficiency (Millán et al., 2002; Guo et al., 2021). As a result, DeepMind introduced the deep Q-network (DQN) algorithm, merging neural networks with Q-learning algorithms. Instead of a Q-table, a neural network stores data, and states and actions are used as the network's inputs, enabling optimal policy development through iterative learning. However, the DQN algorithm mainly functions in discrete environments and struggles with continuous action spaces (Xin et al., 2017). DeepMind then proposed a deep deterministic policy gradient (DDPG) algorithm (Yu et al., 2020), which combined the Actor-Critic framework with DQN and employed a convolutional neural network to simulate the policy function and Q-function, and the output result is a definite action value. Consequently, it overcomes the challenges faced by DRL in high-dimensional or continuous action tasks, making DDPG a more effective path planning algorithm at present. However, the DDPG's random experience replay does not utilize the experience transitions effectively during training (Wei et al., 2022). The algorithm sometimes exhibits slow convergence speed, a low success rate, and inadequate environmental adaptation (Chen et al., 2019; Lin et al., 2021; Liu Q. et al., 2021).

We proposed a dynamic transition priority replay based on multi-dimensional transition priorities. Considering the TD-error, the influence of the Actor network loss function, and the immediate reward on experience transition priority, this method enhances the rationality of experience transition priority computation. It ensures the comprehensive learning of high-value experience transitions and sidesteps the learning of low-value transitions. This method optimizes the use and balance of experience transitions, hastening network convergence. The main contributions of this study are as follows:

(1) To enhance the real-time performance of priority calculation, one should compute the experience transition priority using the Actor network loss function. This calculation should be based on immediate rewards to minimize the frequent sampling of experience transitions with low immediate rewards (e.g., robots distant from the goal point or positioned at the corners of the edges) but with a significant TD-error.
(2) The priority, determined by the immediate reward, TD-error, and Actor network loss function, is integrated using information entropy. This integration serves as the final priority for experience transition, which more accurately reflects the value of experience transition for robot learning.
(3) Positive experience transitions are defined, and their priorities are adjusted based on the training process to enhance the balance of the sampled experience transitions.
(4) The efficacy of the proposed method for robot path planning, based on the DDPG algorithm, is validated. Experimental results demonstrate that the training effect of the proposed method surpasses that of prioritized experience replay (PER) in all types of environments. In addition, it exceeds other state-of-the-art methods and significantly boosts path planning success rates.

## 2 Analysis of experience replay adopted in DDPG

### 2.1 DRL-based path planning formulation

The robot interacts with an environment in DRL-based path planning. The robot observes the state *s*_*t*_∈*S* at each time step *t* and selects action *a*_*t*_∈ 𝒜 based on its policy *a*_*t*_~π(*a*|*s*_*t*_). The robot then receives an immediate reward *r*_*t*_ and moves to the state *s*_*t*+1_∈*S*. The cumulative reward from each time step *t* in an episode is $R_{t}=\sum_{i=t}^{\infty }\gamma^{t}r_{i}$, where γ∈[0, 1) is a discount rate. DRL-based path planning aims to identify the policy π^*^ that maximizes the cumulative reward defined in equation (1).


(1) $$\begin{array}{l} V_{\pi }\left(s\right)=\left[\overset{\infty }{\underset{t=0}{\sum }}\gamma^{t}r\left(s_{t},\pi \left(s_{t}\right)\right)|s_{0}=s\right] \end{array}$$

The *Q*-function under the policy π is defined by equation (2).


(2) $$\begin{array}{l} Q_{\pi }\left(s,a\right)=\left[\overset{\infty }{\underset{t=0}{\sum }}\gamma^{t}r\left(s_{t},a_{t}\right)|s_{0}=s,a_{0}=a\right] \end{array}$$

The *Q*-function under the optimal policy π^*^, denoted Q^*^ as equation (3), satisfies the Bellman optimality equation.


(3) $$\begin{array}{l} Q^{*}\left(s,a\right)=r\left(s,a\right)+\gamma \underset{s^{{}^{\prime }}\in S}{\sum }T\left(s,a,s^{{}^{\prime }}\right)\underset{a^{{}^{\prime }}\in 𝒜}{\max}Q^{*}\left(s^{{}^{\prime }},a^{{}^{\prime }}\right) \end{array}$$

### 2.2 Network structure of DDPG

DDPG uses the Actor-Critic framework, which comprises four networks: the Actor current network, the Actor target network, the Critic current network, and the Critic target network. During the path planning process, the Actor current network outputs the action *a*_*t*_ based on the state *s*_*t*_, receives the environment reward *r*_*t*_ after executing *a*_*t*_, and the robot moves to the next state *s*_*t*+1_. The experience transition [*s*_*t*_, *a*_*t*_, *r*_*t*_, *s*_*t*+1_] generated by the robot–environment interaction is stored in the experience pool. The Actor target network selects the next optimal action *a*_*t*+1_ by *s*_*t*+1_. The current *Q*-value *E*_π_[*Q*(*s*, π(*s*))] and target *Q*-value $E_{\pi^{*}}\left[Q\left(s,\pi^{*}\left(s\right)\right)\right]$ of the current network and target network are determined. TD-error represents their difference. In the training process of the network model, network parameters are updated by minimizing the loss function *L*(θ^*Q*^), defined as the mean value of the TD-error of small batch transitions, and the calculation method is shown in Eqs (4) and (5).


(4) $$\begin{array}{l} L\left(\theta^{Q}\right)=\frac{1}{N}\underset{i}{\sum }{\left(y_{i}-Q\left(s_{i},a_{i}|\theta^{Q}\right)\right)}^{2} \end{array}$$

(5) $$\begin{array}{l} y_{i}=r_{i}+\gamma Q^{{}^{\prime }}\left(s_{i+1},\mu^{{}^{\prime }}\left(s_{i+1}|\theta^{\mu^{{}^{\prime }}}\right)|\theta^{Q^{{}^{\prime }}}\right) \end{array}$$

where *y*_*i*_ is the expected target *Q*-value to make it closer and closer to the expectation of the target network; *N* is the small batch transition size; *r*_*i*_ is the reward of the environment after the robot performs the action *a*_*i*_; θ^μ^′ is the parameters of the Actor target network, θ^*Q*^ and θ^*Q*^′ are the parameters of Critic current network and Critic target network, respectively. The calculation equations of θ^*Q*^′ and θ^μ^′ are shown in (6) and (7), respectively.


(6) $$\begin{array}{l} \theta^{Q^{{}^{\prime }}}\leftarrow \tau \theta^{Q}+\left(1-\tau \right)\theta^{Q^{{}^{\prime }}} \end{array}$$

(7) $$\begin{array}{l} \theta^{\mu^{{}^{\prime }}}\leftarrow \tau \theta^{\mu }+\left(1-\tau \right)\theta^{\mu^{{}^{\prime }}} \end{array}$$

**Figure 1:**
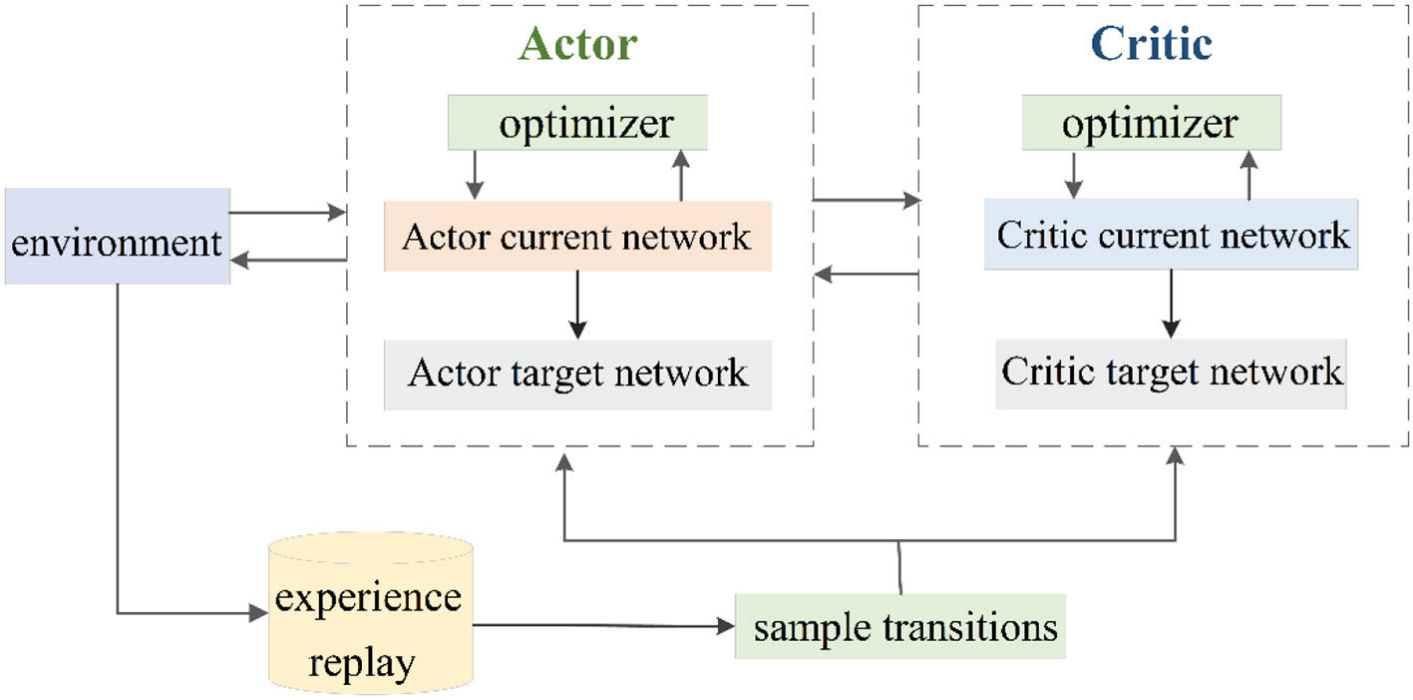
Schematic diagram of the DDPG network.

DDPG target network adopts soft update mode and updates parameters by slowly tracking the learned current network. The advantages of this method are that the target network's parameters change little, the gradient of the current network is relatively stable in the training process, and the stability of the whole algorithm in the learning process is guaranteed. Figure 1 depicts the DDPG network structure (Dong and Zou, 2020).

### 2.3 Prioritized experience replay in DDPG

Random experience replay is an experience transition sampling method originally introduced by the DRL algorithm. Experience transitions are stored in an experience pool, and transitions are selected randomly for training the neural network. This method disrupts the temporal correlation between experience transitions, addressing the issue of non-reusable experience transitions and consequently accelerating the robot's learning process. However, random experience replay utilizes a uniform random sampling method that overlooks the significance of diverse experience transitions in robot learning. This oversight can lead to underutilizing valuable experience transitions and affect the algorithm's training efficiency (Sinha et al., 2022). To address this, DeepMind introduced the PER, which ranks experience transitions based on the absolute value of the TD-error. A larger TD-error indicates a higher value of the experience for robot learning, while a smaller TD-error suggests a lower value. This method allows robots to concentrate on high-value experience transitions, maximizing the use of such experience transitions and enhancing learning speed (Li et al., 2022). PER has advanced the random experience replay method in two significant ways: one refines the sampling probabilities of experience transitions. Equation (8) indicates that the specific method is employed to make the sampling probability *P*(*i*) of the transition *i* proportional to the absolute value of TD-error |δ_*i*_|.


(8) $$\begin{array}{l} P\left(i\right)=\frac{p_{i}^{\alpha }}{\underset{}{\sum_{k}}p_{k}^{\alpha }} \end{array}$$

(9) $$\begin{array}{l} p_{i}=|\delta_{i}|+\epsilon \end{array}$$

where ϵ is constant, mainly to ensure that each experience transition is sampled with a non-zero probability; *p*_*i*_ is the priority of the i-th experience transition; α is a parameter used to control the priority; α = 0 indicates that all transitions are uniformly sampled.

**Figure 2:**
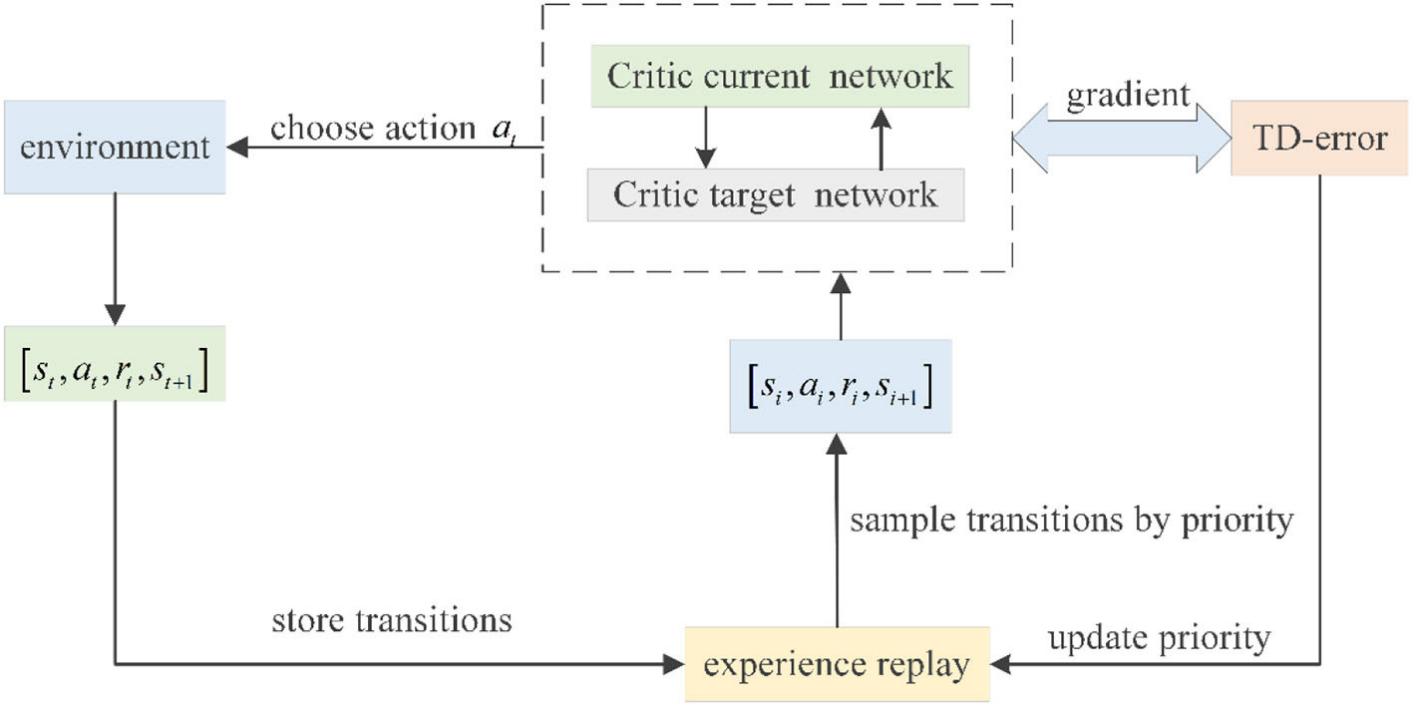
PER in DDPG.

Second, the importance of the sampling method is adopted. The distribution of experience transitions is altered because PER tends to sample experience transitions with a high TD-error value. PER employs significance sampling to correct the significance weights of the transitions and eliminate the bias caused by this method. This ensures that each transition has a different probability of being selected and that the algorithm converges to the same outcome. Equation (10) calculates the weight *w*_*i*_ and applies it to the loss function *L*(θ^*Q*^).


(10) $$\begin{array}{l} w_{i}={\left(\frac{1}{N}\cdot \frac{1}{P\left(i\right)}\right)}^{\beta } \end{array}$$

(11) $$\begin{array}{l} L\left(\theta^{Q}\right)=\frac{1}{N}\underset{i}{\sum }w_{i}{\left(y_{i}-Q\left(s_{i},a_{i}|\theta^{Q}\right)\right)}^{2} \end{array}$$

where β determines the extent to which the influence of PER on the convergence result should be offset. If β = 1, all transitions should be evenly sampled, and the influence should be completely offset. Figure 2 shows the PER process adopted in DDPG.

### 2.4 Problem analysis of PER

Although the incorporation of PER in DDPG enhances the use of experience transitions, the prioritization of these transitions relies exclusively on TD-error, neglecting the impact of other factors on this prioritization (Novati and Koumoutsakos, 2019). It diminishes the robot's efficiency in continuous state-action space, resulting in suboptimal or random behavior (Oh et al., 2007; Fujimoto et al., 2020). Numerous researchers have conducted comprehensive studies to address this concern. Cicek et al. (2021) employed KL divergence (KLPER) for batch prioritization of experience replay. They measured the discrepancy between the batch generation policy and the most recent policy using the KL divergence of a multivariate Gaussian distribution with a mean of zero. Han et al. (2021) introduced the regularly updated deterministic policy gradient algorithm, which organizes experience transitions in block form within the experience pool. This arrangement enabled alternating exploration and learning processes, enhancing the efficacy of experience transition use. Xu et al. (2022) presented the DDPG algorithm with averaged state-action estimation (Averaged-DDPG), calculating action rewards by averaging previously learned *Q*-value estimates, stabilizing the training trajectory, and refining the algorithm's performance. Cao et al. (2019) integrated TD-error, *Q*-value, and data volume, emphasizing varying importance indicators during different neural network training phases and flexibly tuning the weight of each marker to realize adaptive experience transition importance assessment. Li et al. (2021) introduced an internal curiosity module to provide internal rewards for the robot's training phase. These were combined with external rewards from environmental feedback and paired with PER and transfer learning to elevate path planning success rates and hasten convergence. As DRL algorithms targeting transition utilization efficiency have made strides, issues such as low convergence rates and inadequate experience with transition use persist.

**Figure 3:**
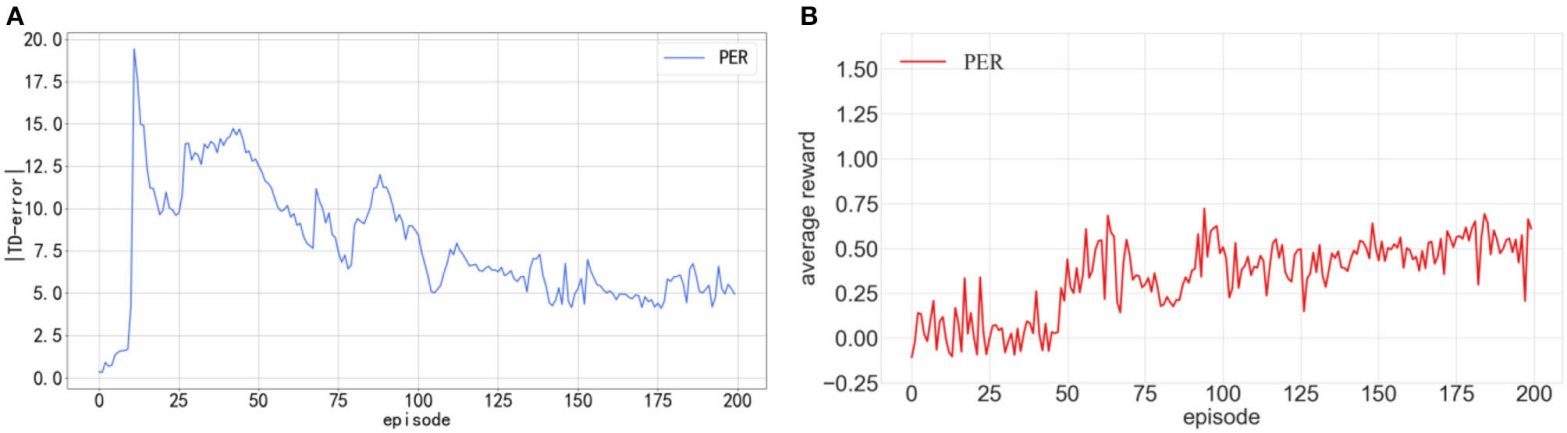
**(A)** Shows the curves of |TD-error| in the training process of DDPG. **(B)** Shows average reward in the training process of DDPG.

We conducted 200 episodes of tests on the DDPG algorithm integrated with PER. The average reward value achieved by the robot after each training episode was recorded. These findings are depicted in Figure 3, where the *x*-axis signifies the count of training episodes, the *y*-axis denotes the average reward value, and the continuous line represents the average value of 10 experimental episodes. The average reward value was determined by dividing the collective reward by the number of robotic movement steps. As illustrated in Figure 3A, an increasing trend in the TD-error curve is evident after 80 training episodes, accompanied by a languid convergence rate. Figure 3B shows that the mean reward consistently remains minimal during training, oscillating ~0.5 in the latter stages, indicating subpar training outcomes. The following two primary considerations drive this observation:

(1) A delay exists in TD-error. The Critic network determines the TD-error associated with the experience transition upon its last sampling. Consequently, if an experience transition's priority is evaluated solely based on its TD-error, a low priority might suggest its diminished relevance to the previous Critic network rather than its standing with the current Critic network.
(2) Sampling transitions with high priority can easily cause an imbalance, which is not conducive to algorithm training. A high-priority transition implies that the Critic network has limited knowledge of that transition, leading to high uncertainty. Consequently, sampling such high-priority transitions can negatively impact the training of Actor networks. For instance, experience transitions in the environment's edge corners are highly valuable, as these areas are rarely explored. Overemphasizing the learning of these transitions can, however, diminish training efficiency.

## 3 The proposed experience replay

**Figure 4:**
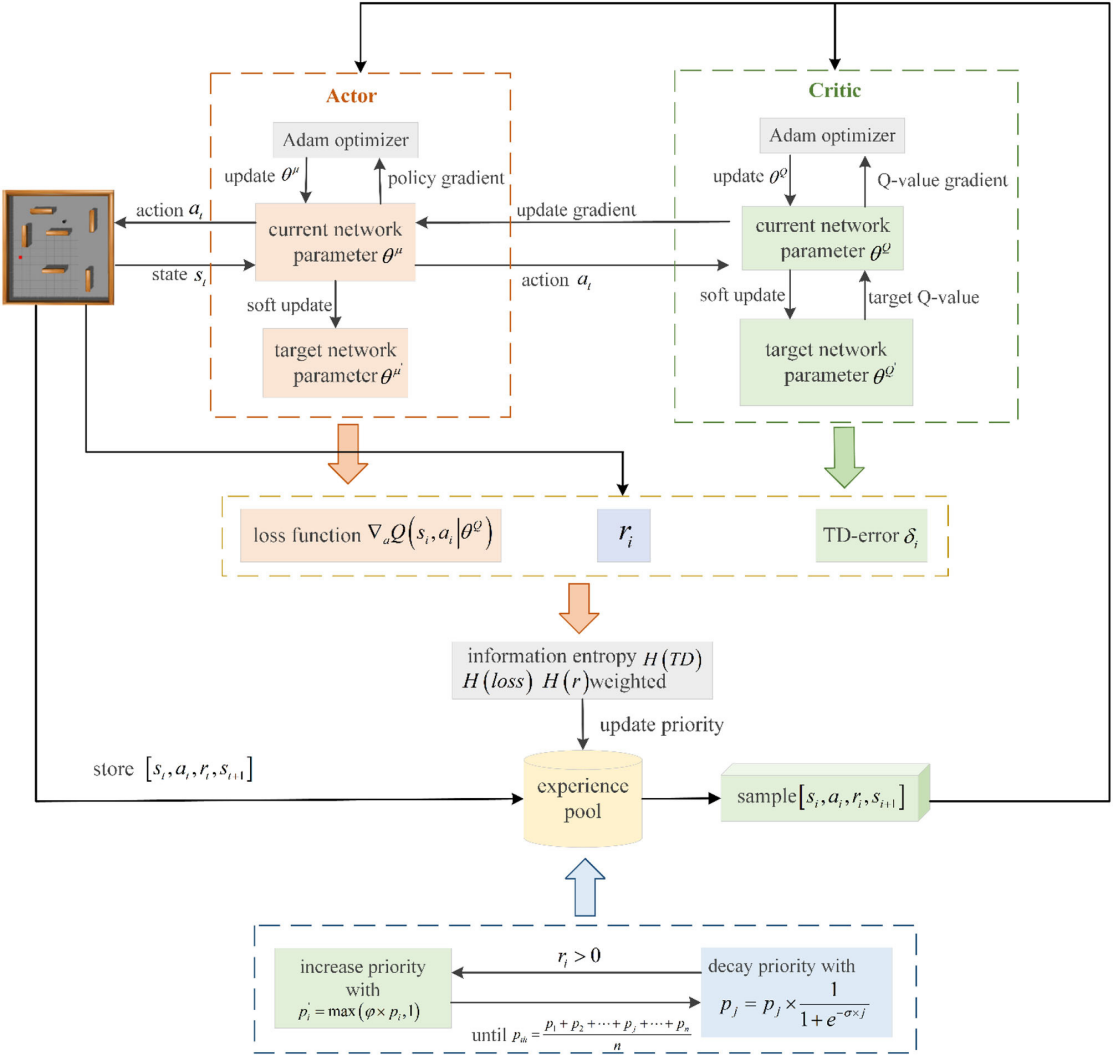
Flow chart of the PER proposed in this study.

We examine the DDPG algorithm's efficiency in utilizing experience transitions by calculating and combining the experience transition priority from multiple dimensions. This method enables the robot to select experience transitions more rationally and effectively during the path planning phase, accelerating the robot's learning process. Priorities of experience transitions are identified sequentially based on the immediate reward, TD-error, and Actor network loss function. These three priorities are combined into a final priority for each experience transition using information entropy weighting. Subsequently, positive experience transitions are characterized, allowing for adaptive adjustment of their priorities throughout the training process. The proposed prioritized experience replay with multi-dimensional priority fusion and priority adjustment (MPFA-PER) is depicted in Figure 4.

### 3.1 Multi-dimensional priority calculation of transitions

For the i-th experience transition [*s*_*i*_, *a*_*i*_, *r*_*i*_, *s*_*i*+1_] in the experience pool, the priority based on immediate reward $p_{i}^{r}$, the priority based on TD-error $p_{i}^{TD}$, and the priority based on Actor network loss function $p_{i}^{loss}$ are defined, respectively. The calculation methods are shown in Eqs (12) through (14).


(12) $$\begin{array}{l} p_{i}^{r}=|r_{i}| \end{array}$$

(13) $$\begin{array}{l} p_{i}^{TD}=|\delta_{i}| \end{array}$$

(14) $$\begin{array}{l} p_{i}^{loss}=|\nabla_{a}Q\left(s_{i},a_{i}|\theta^{Q}\right)| \end{array}$$

where δ_*i*_ is the TD-error calculated from the i-th transition in the experience pool; $\nabla_{a}Q\left(s_{i},a_{i}|\theta^{Q}\right)$ is the loss function of the Actor current network.

Once $p_{i}^{r}$, $p_{i}^{TD}$, and $p_{i}^{loss}$ are determined, they are integrated to form the final priority of the experience transition. Information entropy is utilized to compute the weight coefficients of these three factors. Information entropy represents the probability of discrete random events. It measures the amount of information needed to reduce the uncertainty of events. A higher information entropy indicates that more information is required to dispel the event's uncertainty, and vice versa. The computation of information entropy is presented in Equation (15):


(15) $$\begin{array}{l} H\left(X\right)=-\overset{2}{\underset{i=1}{\sum }}p_{i}\log_{2}\left(p_{i}\right) \end{array}$$

where *X* is the unknown event; *p*_*i*_ is the probability of occurrence of the unknown event.

This study uses Eqs (16) and (17) to calculate the information entropies of immediate reward *H*(*r*), TD-error *H*(*TD*), and the Actor network loss function *H*(*loss*), respectively.


(16) $$\begin{array}{l} H\left(r\right)=-p_{{}_{r>Ravg}}^{}\log_{2}\left(p_{{}_{r>Ravg}}^{}\right)-p_{{}_{r<Ravg}}^{}\log_{2}\left(p_{{}_{r<Ravg}}^{}\right) \end{array}$$

(17) $$\begin{array}{l} H\left(TD\right)=H\left(loss\right)=-p_{r>0}^{}\log_{2}\left(p_{r>0}^{}\right)-p_{r<0}^{}\log_{2}\left(p_{r<0}^{}\right) \end{array}$$

In the training process of the network model, if the immediate reward obtained by the robot exceeds zero, this training is termed positive training, and the resulting experience transition is referred to as a positive experience transition. If not, it is labeled negative training. Where $p_{{}_{r>Ravg}}^{}$ is the probability that the immediate reward is greater than the average reward; $p_{{}_{r<Ravg}}^{}$ is the probability that the immediate reward is less than the average reward; $p_{{}_{r>0}}^{}$ is the probability of active training; $p_{{}_{r<0}}^{}$ is the probability of negative training in all training.

After calculating the three information entropies, the values of the fusion weight coefficients *a*, β and υ of $p_{i}^{r}$, $p_{i}^{TD}$ and $p_{i}^{loss}$ can be determined according to Eqs (18) through (20):


(18) $$\begin{array}{l} a=\frac{H\left(r\right)}{H\left(r\right)+H\left(TD\right)+H\left(loss\right)} \end{array}$$

(19) $$\begin{array}{l} \beta =\frac{H\left(TD\right)}{H\left(r\right)+H\left(TD\right)+H\left(loss\right)} \end{array}$$

(20) $$\begin{array}{l} \upsilon =1-a-\beta \end{array}$$

Based on the estimated weight coefficients, the multi-dimensional transition information is derived by fusing $p_{i}^{r}$, $p_{i}^{TD}$ and $p_{i}^{loss}$. Each experience transition's priority is determined *p*_*i*_ as illustrated in equation (21):


(21) $$\begin{array}{l} p_{i}=\left(a\times p_{i}^{r}+\beta \times p_{i}^{TD}+\upsilon \times p_{i}^{loss}\right)+\epsilon \end{array}$$

where ϵ is minimal constant.

The numerical settings from PER were adopted, and a value of 0.05 was used. When the $\left(a\times p_{i}^{r}+\beta \times p_{i}^{TD}+\upsilon \times p_{i}^{loss}\right)$ of a transition reaches zero, the omission of that transition can be prevented, thereby assigning it a probability to be sampled for training.

### 3.2 Priority increasing of positive transitions

The transition experience with a high absolute value of TD-error in the experience pool suggests a significant discrepancy between the *Q*-values of the Critic current network and the Critic target network, indicating substantial learning potential. Consequently, prioritizing the replay of such experience transitions can swiftly enhance the robot's learning capability. However, solely considering the TD-error during training can neglect the significance of immediate rewards. Experience transitions generated when the robot is positioned at the edge of the environment, known as edge experience transitions, can be excessively used, leading to network overfitting. Experience transitions with successful outcomes or high rewards are termed “positive experience transitions,” which are crucial for robot learning. Sampling more positive experience transitions can expedite the algorithm's convergence and effectively mitigate overfitting. Thus, to prioritize positive experience transitions in experience replay, their priorities are increased based on the priority of experience transitions determined by Eq. (21) as depicted in Eq. (22). The priority of other experience transitions remains unaltered.


(22) $$\begin{array}{l} p_{i}^{{}^{\prime }}\text{=max}\left(\varphi \times p_{i},1\right) \end{array}$$

where φ is a constant.

**Figure 5:**
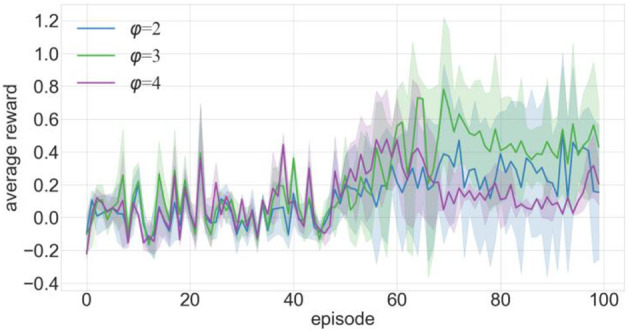
Comparison of training effects of increasing priority using various settings.

**Figure 6:**
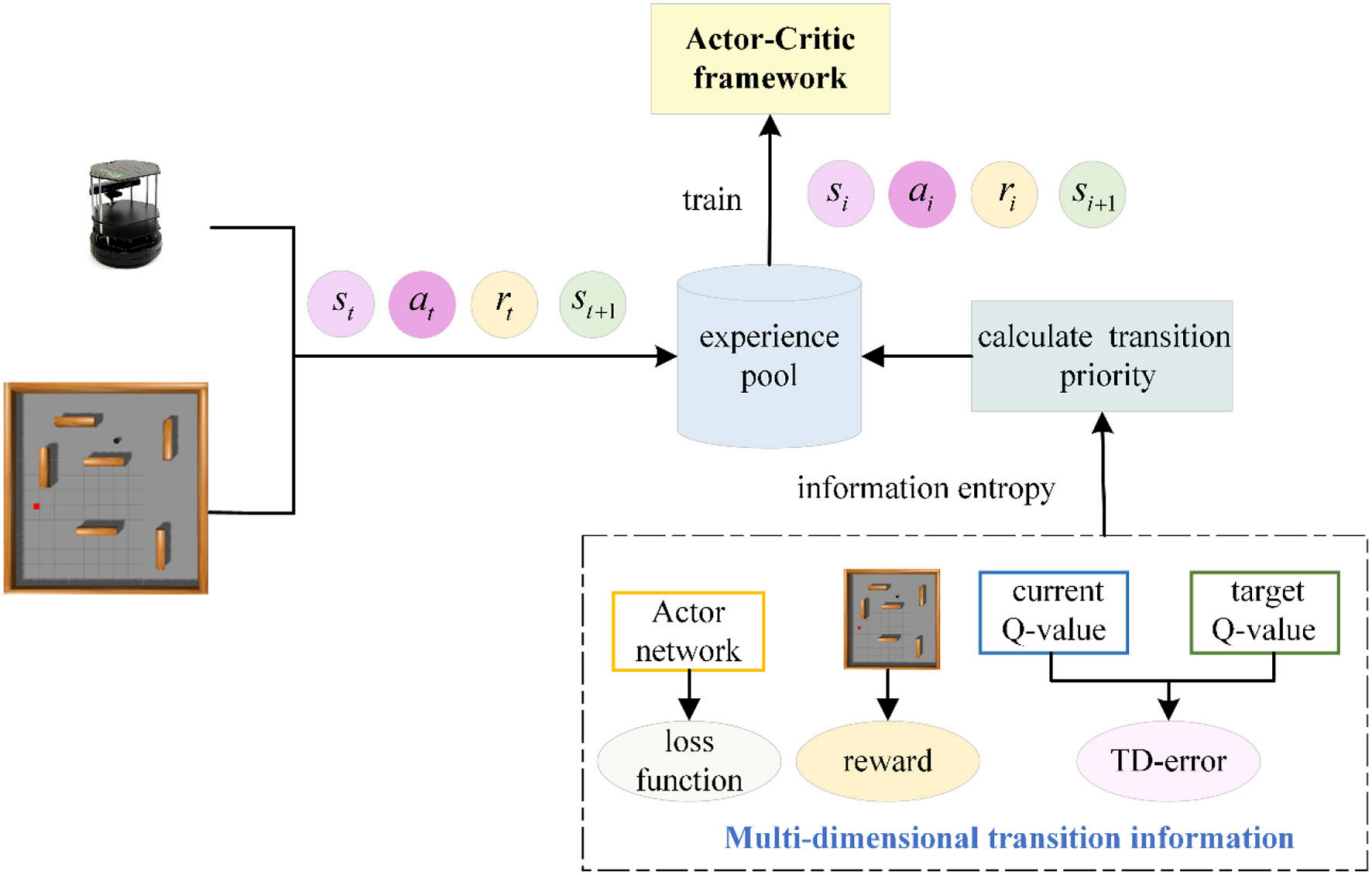
Schematic diagram of multi-dimensional priority calculation of transition.

We conducted 100 episodes of testing for φ =2, 3, and 4 and recorded the average reward value obtained by the robot after each training episode, as shown in Figure 5. In this figure, the *x*-axis is the number of training episodes, the *y*-axis is the average reward value after one training episode, and the solid line is the average of 10 experimental runs. The average reward value is determined by dividing the total reward by the number of steps the robot takes. Figure 5 indicates that the average rewards are roughly similar from episodes 0 to 50. However, as training progressed, the mean reward at φ =4 decreased, while the mean reward at φ =3 increased more than in either of the other two cases. Based on the results of the experiments, it was discovered that setting the value of φ =3 yielded the best performance. Figure 6 depicts the calculation process for the proposed experience transition priority.

### 3.3 Priority decay of positive transitions

In the training process of the DDPG algorithm, high-priority experience transitions prove more beneficial for the algorithm's training. A balance in transitions enhances the adaptive ability of the trained algorithm. Thus, even low-priority transitions should be sampled and learned to maintain this balance. Additionally, to rapidly assimilate the most recent high-priority experience transitions from robot interactions with the environment, updating the priority of existing positive experience transitions in real time is essential. Supposing that a positive experience transition of sampling is *T*_*j*_, its priority is *p*_*j*_, and the priority of the experience transition of the same batch of sampling is expressed as *p* = (*p*_1_, *p*_2_, ···*p*_*j*_···, *p*_*n*_). After the training of this batch of transitions is completed, the priority *p*_*j*_ is attenuated exponentially based on the attenuation factor σ as shown in equation (23).


(23) $$\begin{array}{l} p_{j}=p_{j}\times \frac{1}{1+e^{-\sigma \times j}} \end{array}$$

After several decreases, *p*_*j*_ gradually approach 0, resulting in the transition not being sampled again in the subsequent training process. In order to avoid this problem, a threshold *p*_*th*_ is defined, which allows the priority to reduce when the priority of the transition is greater than *p*_*th*_; otherwise, the decrease stops. *p*_*th*_ is calculated as shown in equation (24):


(24) $$\begin{array}{l} p_{th}=\frac{p_{1}+p_{2}+\cdot \cdot \cdot +p_{j}+\cdot \cdot \cdot +p_{n}}{n} \end{array}$$

After the transition priority decreases, the sampling probability is computed using Equation (8).

### 3.4 Algorithm descriptions

Algorithm 1 represents the detailed description of the proposed algorithm.

**Algorithm 1:**
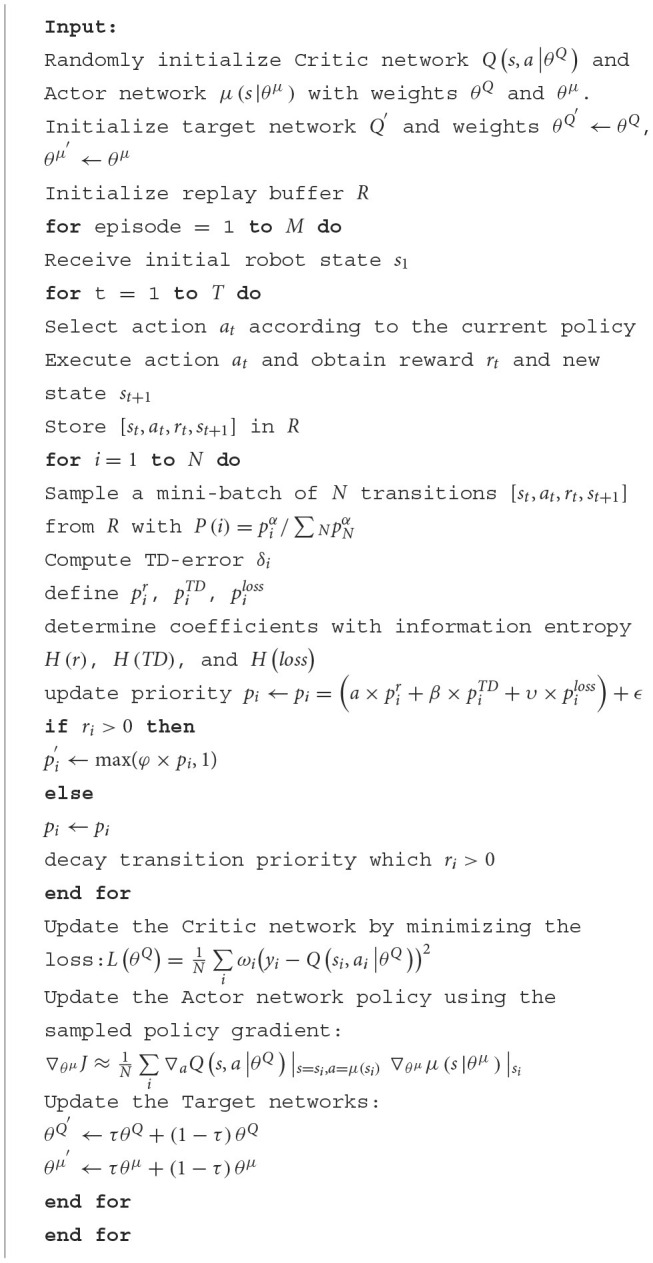
**Prioritized experience replay via multi-dimensional transition priority fusion**.

## 4 Experimental results and analysis

### 4.1 Experimental setting

**Figure 7:**
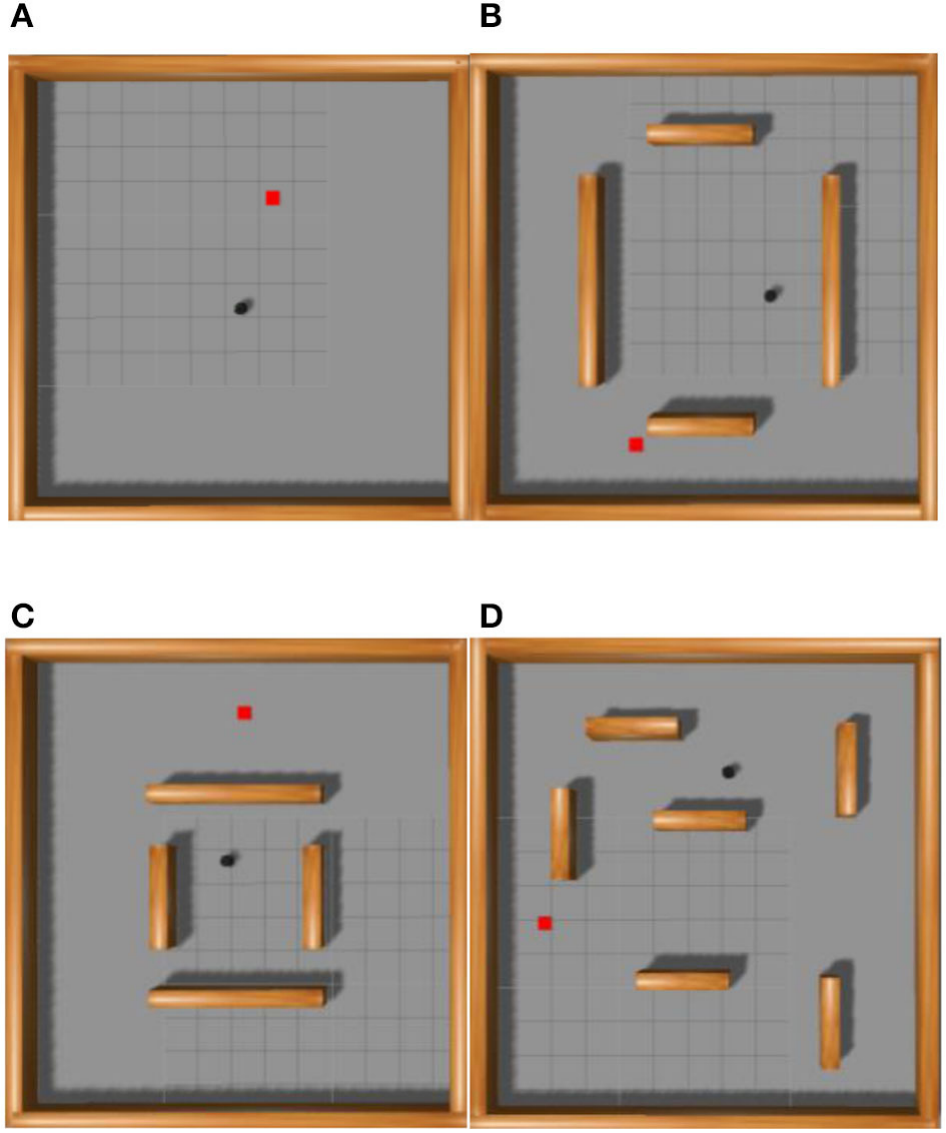
Schematic diagram of simulation environment used to train the path planning algorithm of robot. **(A)** Indicates the environment where there are no obstacles, **(B)** indicates the environment where obstacles are far apart, **(C)** indicates the environment where obstacles are close apart, and **(D)** indicates the environment where obstacles are dense.

The experiment utilized the Gazebo simulation platform, selected the PyTorch framework, and employed the robot operating system for information transmission. The experimental environment measured 10 × 10 m, as depicted in Figure 7. The robot's frontal field of view was set to 180 degrees, and it featured 18 distance measurements with a resolution of 10 degrees each. The robot model resembled a black TurtleBot, with red squares indicating the target points and cuboids denoting the obstacles. The starting and target points for the robot's movement were randomly positioned within the entire map, ensuring no overlap with the obstacles. The algorithms proposed in this study conducted 100, 200, 300, and 400 episodes of training experiments in the environments illustrated in Figures 7A–D, respectively. Figure 7A shows a square environment devoid of obstacles, which trains the robot in path planning within a confined setting. Figure 7B shows four cuboid obstacles, spaced widely apart, to the environment in Figure 7A to cultivate the robot's avoidance capabilities in a restricted area. The gap between the four obstacles narrows in Figure 7C to intensify the robot's training in obstacle avoidance within tighter spaces. In Figure 7D, numerous obstacles supplement the environment from Figure 7A to train the robot's path planning ability in intricate settings.

During the training of path planning algorithms, the reward attained by the robot's interaction with its environment in a training episode serves as a crucial metric for evaluating algorithmic performance. If the reward is high and consistent, the episode's training outcome is deemed satisfactory; if not, it is considered unsatisfactory. As expressed in Eq. (25), the reward function can determine the reward the robot accrues during training. Upon reaching the target point, the robot receives a reward of 10. A collision with an obstacle incurs a penalty of −5. If the robot neither collides with obstacles nor attains the target point, the distance variance between the robot and the target at times *t*−1 and *t* is calculated as a reward, motivating the robot to navigate closer to the target in subsequent moves.


(25) $$R=\{\begin{array}{c} 10\\ -\text{5}\\ \varepsilon_{p}\times \left(d_{t}-d_{t-1}\right) \end{array}\;\begin{array}{c} \text{robot reaches the goal}\\ \text{robot collides}\\ otherwise \end{array}$$

where ε_*p*_ is the amplification factor; *d*_*t*_ and *d*_*t*−1_ are the distance between the robot and the target point at time *t*−1 and time *t*, respectively.

In order to ensure the robot completes the path planning algorithm training, the following termination conditions must be set: (1) If the robot reaches the specified target point without collision, a new training episode is initiated; (2) if the robot collides with obstacles or exceeds the maximum number of steps, the current training episode is terminated and a new one begins. The rationale for setting a maximum step count is to prevent ineffective training. The experimental parameters are listed in Table 1.

**Table 1:** Setting of experimental parameters.

**Parameter**	**Value**
Learning rate of critic network	0.0001
Learning rate of actor network	0.0001
Reward discount rate γ	0.99
Soft update parameter τ	0.01
Total number of episodes	1,000
Experience pool capacity	100,000
Batch size	256

### 4.2 Results and analysis of algorithm training

This section presents experiments designed to address the following questions:

(1) Can multi-dimensional transition priority calculation enhance the path planning performance of the DDPG algorithm?
(2) Can the performance of the DDPG algorithm in path planning be improved by dynamically modifying the transition priority?

#### 4.2.1 Validity test of priority calculation of multi-dimensional transitions

**Figure 8:**
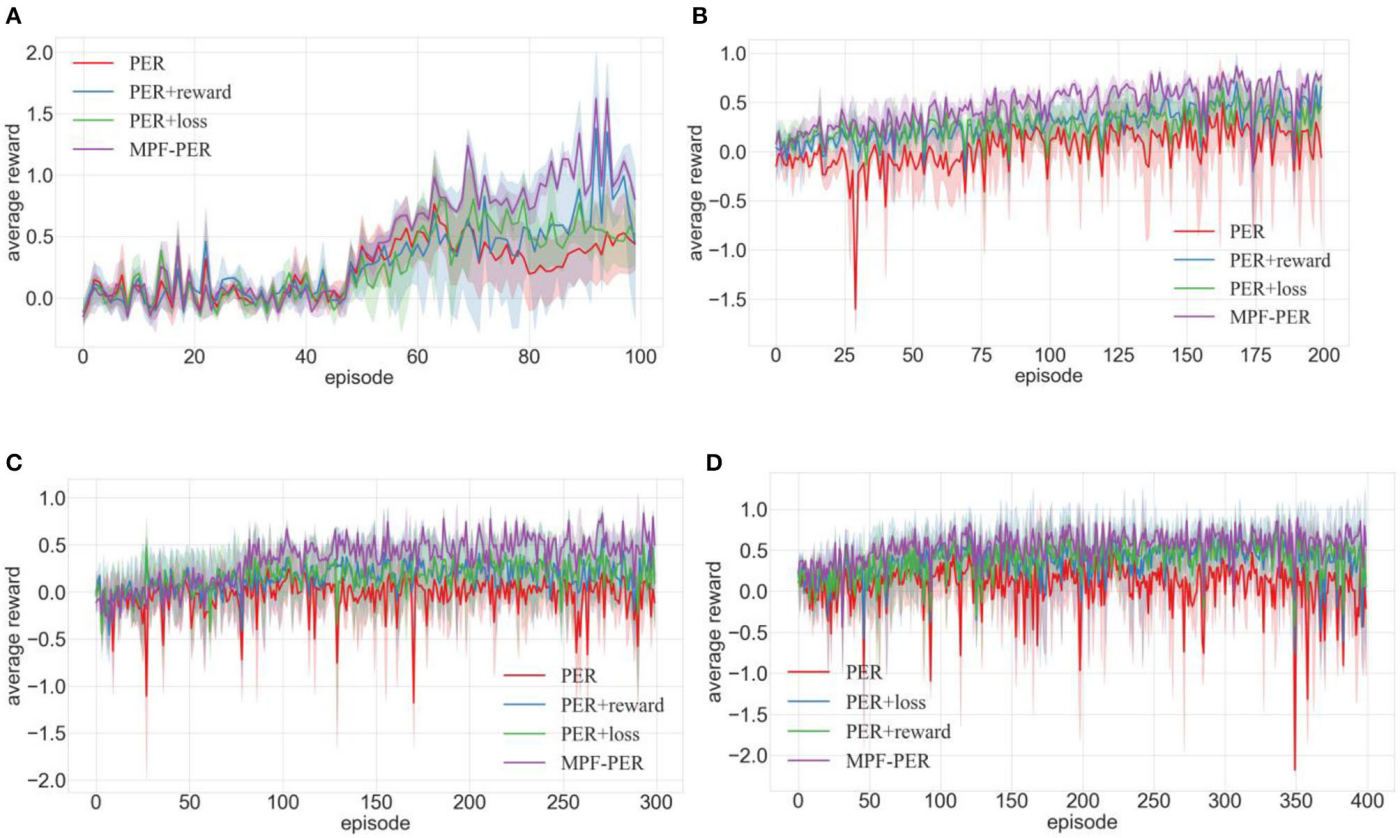
Comparison of training effects of DDPG algorithm with different priority calculation methods in different environments (**A–D** are the training results in the four training environments shown in Figure 7).

In order to address the first question, we independently used the immediate reward, TD-error, and Actor loss function. The multi-dimensional Priority Fusion PER (MPF-PER) was employed to determine the priority of the experience transition. This was incorporated into the DDPG algorithm's training process. In the environments depicted in Figures 7A–D, training was conducted for 100, 200, 300, and 400 episodes, respectively. The average reward value obtained by the robot after each training episode was documented, with the results presented in Figures 8A–D. In this study, the *x*-axis denotes the number of training episodes, while the y-axis signifies the average reward value obtained after a single episode. The data in Figure 8 represent the average values from 10 experiments. The average reward value is the total reward divided by the robot's number of movement steps.

The immediate reward's impact on the experience transition priority calculation was assessed. The immediate reward was incorporated into the PER to determine its efficacy during training. Results are illustrated in Figure 8 with the blue line (TD-error + reward), which is contrasted against the red line representing PER. Figure 8A reveals that the training efficacy of PER with the immediate reward surpassed that of standalone PER after 75 episodes, despite a minor decline in PER experience transitions. Figure 8B indicates that the PER with the immediate reward stabilized within 50 episodes with minimal fluctuation and consistently outperformed the PER. Though PER stabilized after 75 episodes, its fluctuations were pronounced, suggesting that robots either missed their target or encountered obstacles often during training, resulting in limited path planning success. Figures 8C, D exhibit that the training effect of PER with immediate reward consistently surpassed the average reward of PER. This indicated that incorporating the immediate reward into the priority calculation method significantly influences the training process.

The influence of the Actor network loss function on experience transition priority calculation was also investigated. It was integrated into the PER to ascertain the loss function's role during training. The green line (TD-error + loss) represented the outcome in Figure 8 and was primarily contrasted with the red line symbolizing PER. Figure 8A shows that the training effect of PER with a loss function was inferior to that of PER during episodes 50–60, whereas the training effect between episodes 60–70 was comparable. However, after 70 training episodes, the PER with Actor loss function converged and outperformed the standalone PER. In Figures 8B–D, PER enhanced with a loss function demonstrates a superior training effect than PER, indicating that incorporating a loss function can effectively improve the success rate of robot path planning.

We examined the effect of information entropy weighting on three types of information for experience transition priority computation. The MPF-PER algorithm underwent separate training to test the effectiveness of this information entropy weighting. The resulting line, denoted as MPF-PER in Figure 8, was compared to the priority calculation method that incorporated each type of information independently. In Figure 8A, even though the training outcome of MPF-PER resembled that of other algorithms up to the 50th episode, there was a significant increase after the 50th episode, as depicted in Figures 8B–D. This suggested that the integration of the three types of information significantly impacted the training.

In the above experiments, both immediate reward and Actor network loss functions were incorporated into the PER training, highlighting their pivotal role in transition priority computation. The inclusion of information entropy bridged the disparities among the three information types, reflecting the enhanced training effect of the MPF-PER within the DDPG algorithm.

#### 4.2.2 Effectiveness test of adaptive adjustment of experience transition priority

**Figure 9:**
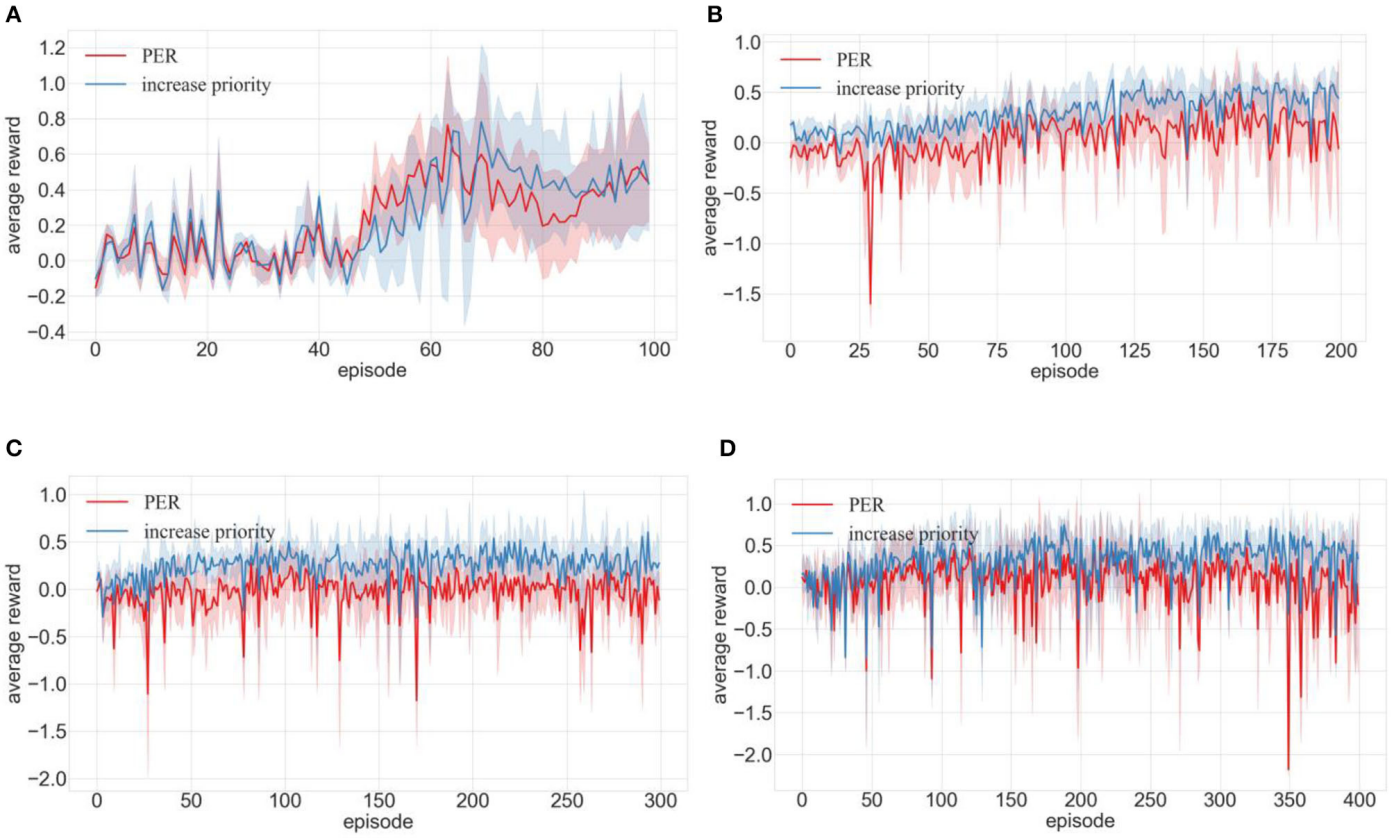
Comparison of training effects of DDPG algorithm integrated with priority increasing and PER in different environments (**A–D** are the training results in the four training environments shown in Figure 7).

**Figure 10:**
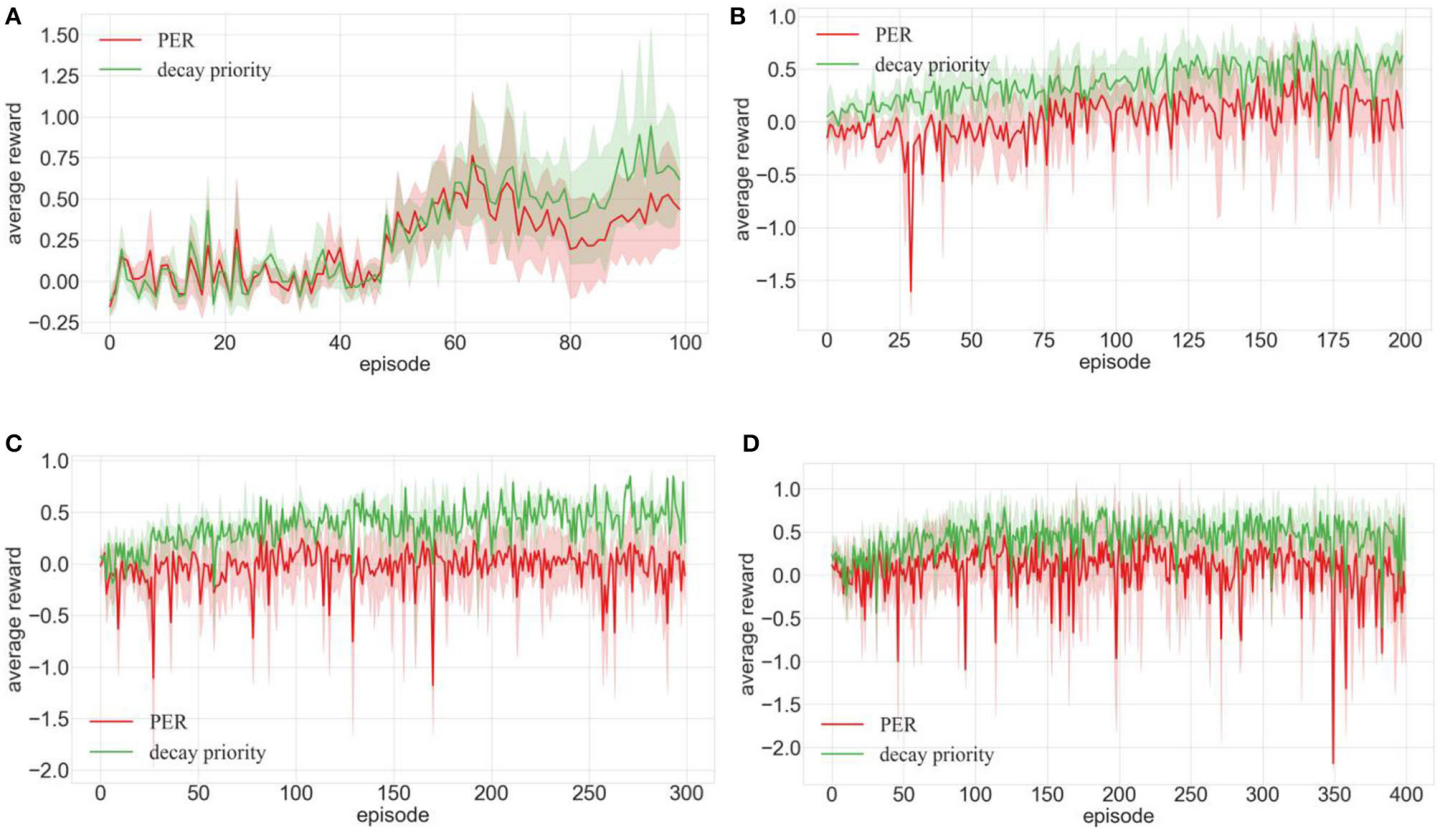
Comparison of training effects of DDPG algorithm integrated with priority decay and PER in different environments (**A–D** are the training results in the four training environments shown in Figure 7).

**Figure 11:**
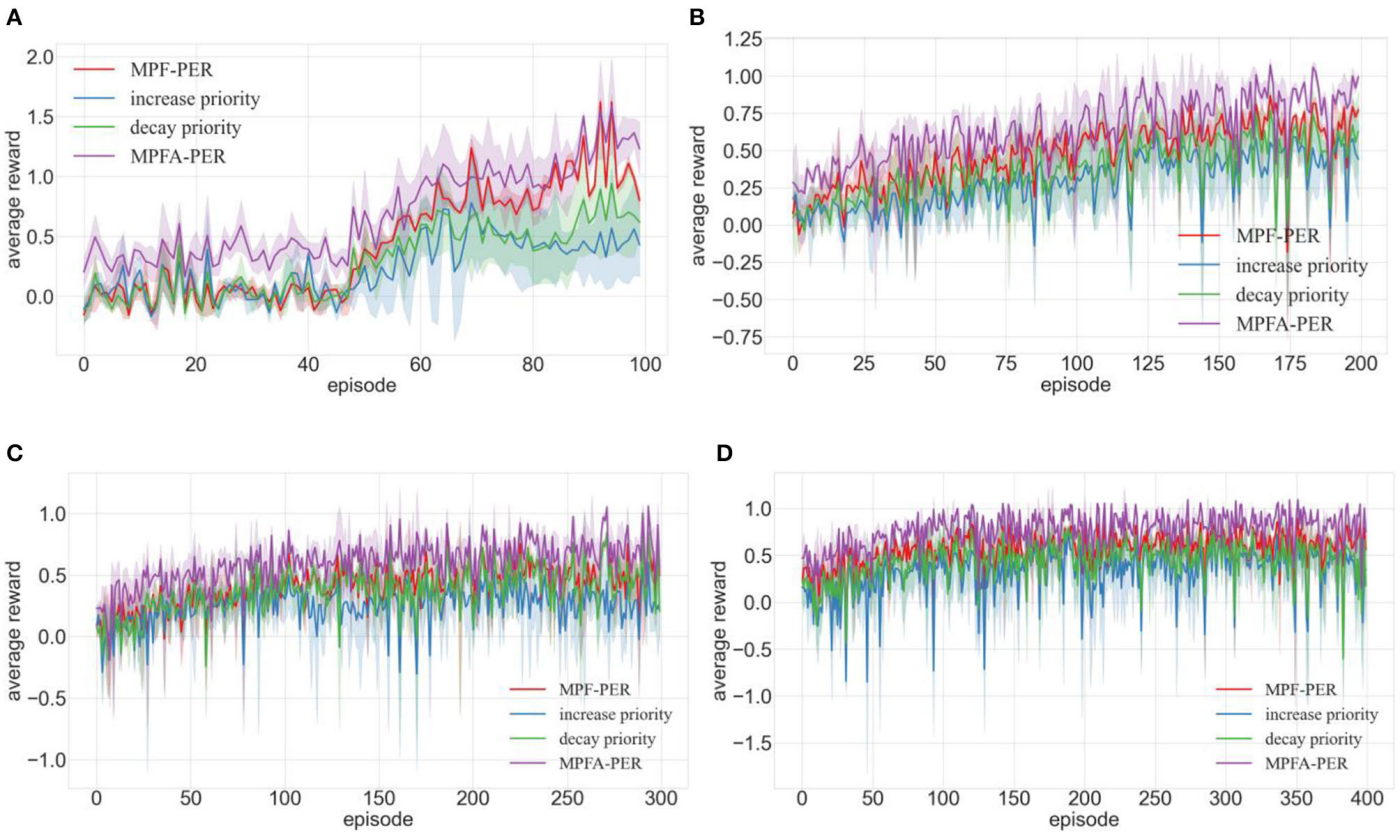
Training of DDPG algorithm integrated with MPFA-PER in different environments (**A–D** are the training results in the four training environments shown in Figure 7).

In response to question 2, we integrated increase priority, decay priority, MPF-PER, and MPFA-PER into the DDPG algorithm. The environment depicted in Figures 7A–D underwent 100, 200, 300, and 400 training episodes, respectively. Figures 9–11 present the average reward values achieved by the robot after each training. The data illustrated in the figures represent the average values of 10 experiments.

##### 4.2.2.1 Effectiveness of priority increasing

Figure 9A shows that even though PER's average reward spikes between episodes 45 and 60, the average reward of PER with increased priority distinctly surpassed that of the standard PER post the 70th episode. Figure 9B shows that the average reward of PER declined drastically during the 27th episode, with the trajectory showing volatility until it stabilized at the 140th episode. This suggested repetitive failures in robot navigation toward the target or frequent obstacles, culminating in training hindrances. However, PER with increased priority attained the zenith of average reward, stabilizing at the 125th episode. Figures 9C, D shows that the average reward of PER with an elevated priority consistently surpassed the regular PER, emphasizing its superior training for positive experience transitions and enhanced robot path planning.

##### 4.2.2.2 Effectiveness of priority decay

Figures 10, 11 display the robot's average rewards across four environments. Figure 11A shows that decay priority mirrors the average reward of increased priority until, after the 90th episode, the average reward of PER with decaying priority notably excels. In Figure 10A, the average reward of PER post the 70th episode significantly outperformed that of standard PER. Despite pronounced fluctuations in the training curve, as depicted in Figures 10B–D, the average reward consistently held a median position, always ahead of the increased priority. Analyzing data from Figures 10, 11 affirmed its superiority over the PER, underscoring the role of decay priority in robot performance enhancement.

##### 4.2.2.3 Effectiveness of MPF-PER

Figure 11 substantiates that the DDPG algorithm fortified with MPF-PER performs better across varied testing environments than its increased and decayed priority counterparts. Introducing MPFA-PER to the DDPG algorithm yielded the most optimal training outcomes for the DDPG algorithm.

The above experimental results show that both increase and decay priorities improve the average reward during the DDPG algorithm training, yielding more stable training results. The substantial fluctuations in the average reward garnered by PER throughout the training process render it unstable. This instability arises from PER's reliance on TD-error for assessing experience transition priority, overlooking the immediate reward's influence during network training. Such an oversight can lead to excessive reliance on edge experience transitions and result in localized optimality. Transitions are prioritized more precisely by considering all three factors—immediate reward, TD-error, and Actor network loss function—integrating them with information entropy. The methodology of decaying the priority of positive experience transitions ensures a balanced sampling of both low-priority experience transitions and those of the latest high priority. This equilibrium in training transitions maintains the algorithm's training more steadfastly. The convergence speed is faster than that of PER, and the success rate of path planning is also higher than that of PER.

### 4.3 Results and analysis of algorithm testing

**Figure 12:**
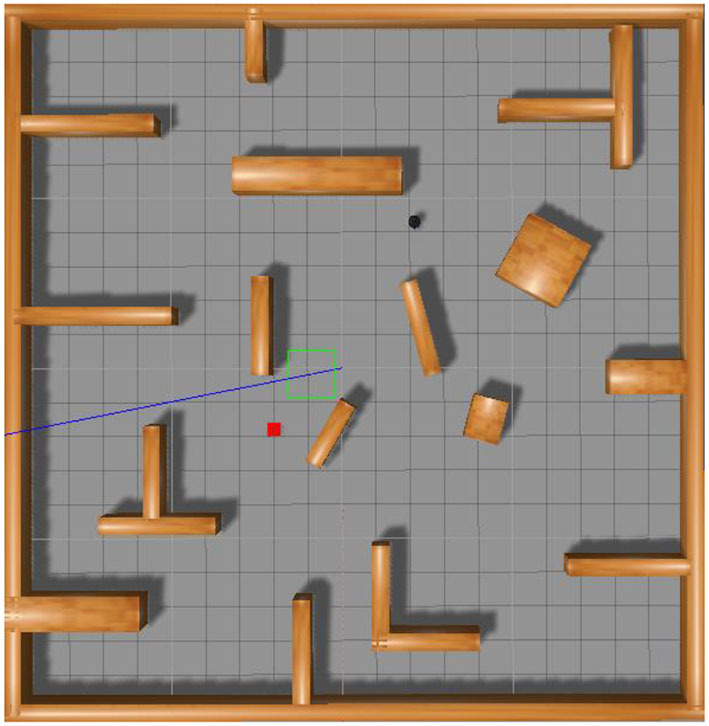
Schematic diagram of the simulation environment used to test the path planning algorithm of robot.

In order to verify the effectiveness and success rate of the proposed algorithm for path planning in unknown environments, we incorporated the increased priority, decay priority, MPF-PER, and MPFA-PER methodologies into the DDPG algorithm. After training, the algorithm underwent testing for 200 episodes in a new unknown simulation environment, as depicted in Figure 12. The black dot represents the robot, the red square signifies the target point, and the brown block objects symbolize obstacles. The obstacle density in the testing environment exceeds that of the training environment.

Table 2 presents the test outcomes of the robot in the environment depicted in Figure 12. When each algorithm component was trained independently, the training effectiveness surpassed that of PER. The proposed algorithm reduced collision instances by 34.48% compared to PER, and the likelihood of the robot successfully reaching the target point increased by 5%. This demonstrated a higher success rate for the path planning algorithm and enhanced safety for the robot during movement. Regarding time consumption, the proposed algorithm's average test duration was 27.3 s, which was 14.15% less than that of PER. This suggested that the algorithm facilitated faster target attainment by the robot, thereby elevating the operational efficiency of the robot.

**Table 2:** Comparison of algorithm performance in the test environment.

**Algorithm**	**Number of collisions**	**Success rate (100%)**	**Average test time (s)**
PER	29	85.5	31.8
TD-error + reward	24	88	30.1
TD-error + loss	25	87.5	30.5
MPF-PER	21	89.5	28.2
Raise priority	22	89	30.3
Decay priority	23	88.5	29.8
MPFA-PER	**19**	**90.5**	**27.3**

The bolded column shows the experimental results of our proposed method, showing that our method is the most effective.

### 4.4 Comparison with other algorithms

We proposed MPFA-PER to evaluate and compare the effectiveness of the prioritized experience replay method with the following methods.

(1) KLPER (Cicek et al., 2021): The mean KL divergence was employed to measure the bias between policies as the prioritized transition standard.
(2) Averaged-DDPG (Xu et al., 2022): DDPG with average state action was estimated.
(3) HVPER (Cao et al., 2019): Calculate transition importance considering TD-error, *Q*-value, and data volume.
(4) DDPG + RAdam (Li et al., 2021): The RAdam algorithm, which incorporated the curiosity algorithm, replaced the neural network optimizer.
(5) MW-MADDPG (Zhao et al., 2023): Transition priority was determined using TD-error and rewards, and after a certain number of transitions, the transition was no longer selected for sampling.

**Figure 13:**
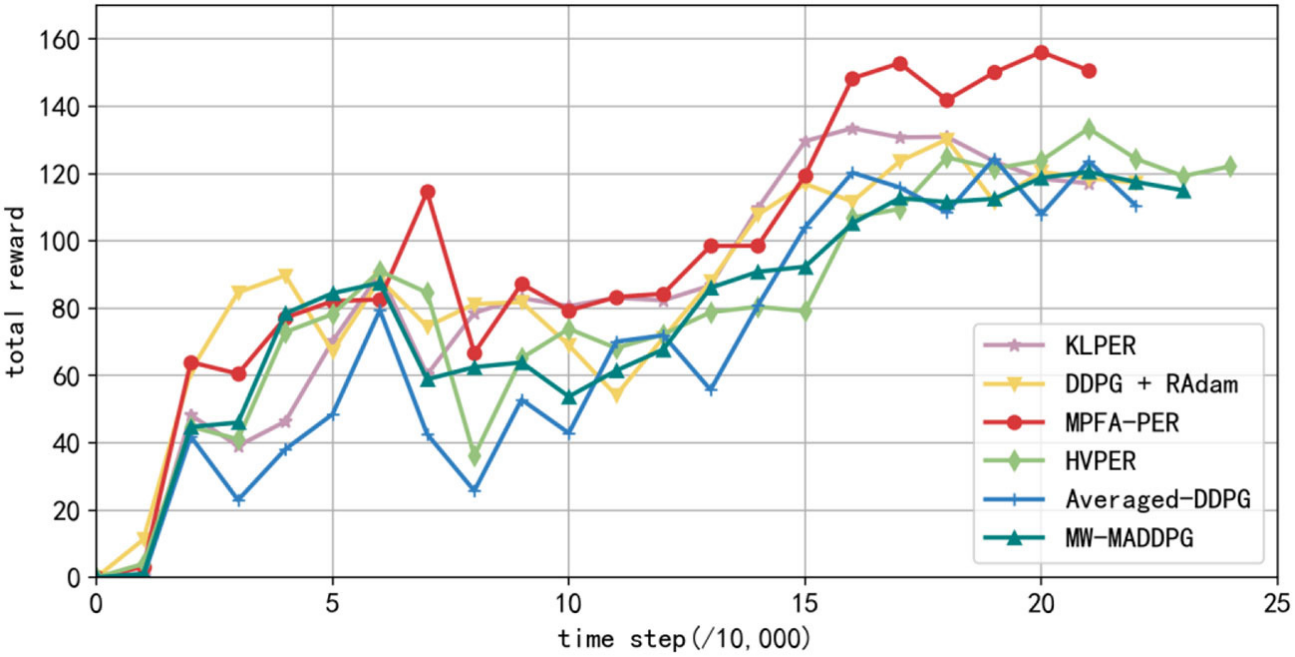
Total reward obtained every 10,000 steps in the training process of different algorithms.

In the simulation environment depicted in Figure 7, each algorithm underwent training for 100, 200, 300, and 400 episodes sequentially. The total rewards secured by the robot every 10,000 steps are depicted in Figure 13. The data presented in Figure 13 represent the average values from 10 experiments. The y-axis showcases the total reward earned by the robot, while the *x*-axis denotes the number of steps taken. The figure illustrates the robot's total reward per 10,000 steps. The total reward for each algorithm has been increasing. However, the reward from the algorithm we proposed remained elevated, achieving convergence after 130,000 steps at the most rapid rate. Beyond 160,000 steps, the reward from the studied algorithm was substantially higher than the others. KLPER showed gradual convergence in path planning, reaching a plateau of ~140,000 steps. However, post-convergence, there was a declining trend in the reward, suggesting an imperfect path. Both DDPG + RAdam and HVPER exhibited similar training effects. DDPG + RAdam reached convergence in ~140,000 steps. Meanwhile, HVPER and MW-MADDPG lag, converging approximately at 160,000 steps. The total reward of the Averaged-DDPG was the least, converging ~150,000 steps.

Table 3 provides details on the training duration and steps for each algorithm. From this table, HVPER has the longest training duration of 2,082 min, whereas MW-MADDPG requires 1,675 min. DDPG + RAdam and Averaged-DDPG have training durations exceeding 1,500 min, but KLPER's duration was shorter at 1,284 min. In particular, the training time for the algorithm we proposed was a mere 994 min, outpacing the others. Regarding the number of training steps, HVPER tops the list with 247,043 steps, followed by MW-MADDPG at 233,047. DDPG + RAdam and Averaged-DDPG exceeded 220,000 steps, with KLPER even fewer at 219,318 steps. The proposed algorithm has the fewest steps at 213,627. Both in terms of duration and steps, the algorithm under investigation outperformed KLPER, Averaged-DDPG, HVPER, DDPG + RAdam, and MW-MADDPG.

**Table 3:** Comparison of training time and steps of different algorithms.

**Algorithm**	**Training time (min)**	**Training steps (step)**
KLPER	1,284	219,318
DDPG + RAdam	1,589	224,048
HVPER	2,082	247,043
Averaged-DDPG	1,522	222,569
MW-MADDPG	1,675	233,047
MPFA-PER	**994**	**213,627**

The bolded column shows the experimental results of our proposed method, showing that our method is the most effective.

To assess the success rate of the path planning algorithm in unfamiliar environments, our proposed and other methods, such as KLPER, Averaged-DDPG, HVPER, DDPG + RAdam, and MW-MADDPG, underwent 200 tests in the simulation environment of Figure 12. Table 4 shows the collision count, success rate, and average consumption time. The success rate of Averaged-DDPG was the lowest at 86%. HVPER marginally improved to 86.5%, but its average duration was the longest at 34.2 s. DDPG + RAdam and Averaged-DDPG shared a similar average time exceeding 30 s, though DDPG + RAdam has a marginally better success rate. MW-MADDPG matched HVPER's 86.5% success rate but had a shorter average time of 32.8 s. KLPER posted an 88% success rate and an average time of 29.4 s. Remarkably, the algorithm we proposed boasted a 90.5% success rate and the shortest average time of 27.3 s. This suggested that, compared with other algorithms, the proposed algorithm ensured that the robot reached its target more swiftly and reliably. Additionally, it has the lowest collision rate, 19 times, marking a reduction of 20.83% relative to KLPER, 24% relative to DDPG + RAdam, 29.63% compared to HVPER and MW-MADDPG, and 34.48% against Averaged-DDPG. This underscored the enhanced safety assurance of the robot during path planning and highlighted the proposed algorithm's superior performance in path planning tasks.

**Table 4:** Comparison of success rate and average test time of different algorithms.

**Algorithm**	**Number of collisions**	**Success rate (100%)**	**Average test time (s)**
KLPER	24	88	29.4
DDPG + RAdam	25	87.5	31.5
HVPER	27	86.5	34.2
Averaged-DDPG	28	86	30.9
MW-MADDPG	27	86.5	32.8
MPFA-PER	**19**	**90.5**	**27.3**

The bolded column shows the experimental results of our proposed method, showing that our method is the most effective.

## 5 Conclusion

We proposed an enhanced method for determining the priority of experience transitions. Experience transitions are further optimized by increasing the priority of positive transitions and utilizing a decay method. This leads to a faster convergence speed in path planning, enabling the robot to reach its goal more safely and efficiently. However, when increasing the priority of positive transitions, a fixed parameter derived from experimental data is employed. The priority calculation can be more accurate if this parameter can be adaptively modified during the training process. The current algorithm focuses solely on scenarios with static obstacles in the environment and neglects situations with dynamic obstacles. Future research will focus on determining how to adjust the parameter more appropriately, mitigating the influence of dynamic obstacles on path planning, and enhancing the efficiency of path planning in intricate environments.

## Data availability statement

The original contributions presented in the study are included in the article/supplementary material, further inquiries can be directed to the corresponding author.

## Author contributions

NC: Methodology, Software, Writing—original draft, Writing—review & editing. PW: Supervision, Writing—review & editing. GZ: Conceptualization, Methodology, Writing—review & editing. CN: Writing—review & editing. EN: Writing—review & editing.
